# Antenna Design and Optimization for 5G, 6G, and IoT

**DOI:** 10.3390/s25051494

**Published:** 2025-02-28

**Authors:** Haleh Jahanbakhsh Basherlou, Naser Ojaroudi Parchin, Chan Hwang See

**Affiliations:** School of Computing, Engineering and the Built Environment, Edinburgh Napier University, Edinburgh EH10 5DT, UK; c.see@napier.ac.uk

## 1. Introduction

In the ever-expanding domain of wireless communication, the continuous evolution of network technologies has underscored the critical role of antenna design and optimization. As we transition from 5G to the emerging era of 6G and the Internet of Things (IoT), the need for highly efficient, adaptive, and multifunctional antennas has become paramount [1,2]. The increasing demand for seamless connectivity, ultra-reliable low-latency communication (URLLC), massive machine-type communication (mMTC), and enhanced mobile broadband (eMBB) necessitate innovative antenna solutions that can cater to diverse and stringent performance requirements. Antennas are no longer mere transmission and reception devices; they serve as fundamental enablers of advanced wireless systems, dictating the efficiency, reliability, and scalability of next-generation networks [3,4]. With the advent of reconfigurable intelligent surfaces (RIS) and metamaterials, antenna technology is poised to revolutionize wireless communication by enabling dynamic beamforming, energy efficiency, and spectrum utilization enhancements [5,6].

The integration of advanced antenna technologies is central to addressing the challenges associated with higher frequency bands, increased data rates, and network densification [7]. In 5G and beyond, Multiple-Input Multiple-Output (MIMO) systems, beamforming techniques, and reconfigurable intelligent surfaces (RIS) have emerged as key solutions to optimize spectral and energy efficiency. Furthermore, the move towards sub-terahertz (THz) and millimeter-wave (mmWave) frequency bands has introduced new complexities in antenna design, including the need for compact, high-gain, and low-loss structures [8]. The convergence of artificial intelligence (AI) and machine learning (ML) in antenna design and optimization is also paving the way for smart, self-adaptive antennas capable of dynamic performance enhancement. The rapid proliferation of IoT applications has further fueled research in miniaturized, energy-efficient, and cost-effective antennas. From smart cities and autonomous vehicles to healthcare monitoring and industrial automation, IoT-driven ecosystems demand antennas that can seamlessly operate across multiple frequency bands while ensuring reliability in highly dynamic environments. The integration of flexible and conformal antennas, alongside novel materials such as graphene and metamaterials, is redefining the landscape of IoT connectivity [9].

This Special Issue focuses on the latest advancements in antenna design and optimization for 5G, 6G, and IoT applications. The contributions presented in this collection explore cutting-edge methodologies, novel architecture, and emerging trends that are shaping the future of wireless communication. Topics range from innovative MIMO and phased-array configurations to the development of highly efficient THz and mmWave antennas, as well as AI-driven optimization techniques. By providing a comprehensive overview of state-of-the-art research, this Special Issue aims to foster new insights and inspire further exploration into next-generation antenna technologies. The research featured here not only highlights significant theoretical and experimental advancements but also underscores the transformative potential of antennas in shaping the future of connectivity.

## 2. Overview of Published Papers

Jahanbakhsh Basherlou et al. (Contribution 1) present a newly designed MIMO antenna array tailored for sub-6 GHz 5G applications incorporating eight compact trapezoidal slot elements with L-shaped CPW feedlines, ensuring wide bandwidth and polarization diversity. The antennas, placed at the smartphone’s peripheral corners, cover 3.2–6 GHz with a compact 75 mm × 150 mm footprint. The array achieves high isolation without decoupling structures and provides a bandwidth of 2800 MHz. It demonstrates excellent radiation efficiency, low ECC (<0.005), and low TARC (<−20 dB). The design is implemented on a single-layer FR4 substrate, optimizing cost and performance. Experimental results closely match simulations, confirming reliability. A compact mmWave phased array is introduced for future 5G/6G applications. The design supports high data rates, efficient connectivity, and future smartphone integration.

Hoang et al. (Contribution 2) present a characterization of near-field impulse responses in a double-slot Vivaldi antenna using electromagnetic (EM) near-field data. Their analysis explores EM energy flow, reorientation, and scattering across different antenna segments. Geometric features of near-field wavefront surfaces aid in evaluating antenna directivity and optimizing structural details. The study introduces partitional far-field response characteristics in frequency and time domains. By simplifying the near-field propagation model, the approach enhances insight into EM propagation in Vivaldi antennas. The proposed methodology identifies dominant EM flow regions and structural impacts on radiation performance. Partitional gain and impulse response characteristics guide structural optimization. The study highlights how localized EM characteristics improve antenna design efficiency. The optimization process is validated through near-field, far-field, and conventional characteristics. This framework is also applicable to other traveling wave antennas for better propagation understanding and design improvements.

Lin et al. (Contribution 3) propose a wideband dual-polarized dipole antenna operating from 1.7 to 3.8 GHz. Traditional 4G dipole antennas struggle with impedance matching and stable radiation patterns in the 5G sub-6 GHz band. To address this, a connected-ring-shaped metasurface structure functions as an artificial magnetic conductor (AMC). The AMC stabilizes radiation patterns without increasing antenna height. The measured results confirm an 80.7% impedance bandwidth (1.7–4.0 GHz). The antenna maintains a stable realized gain of 7.0 ± 1.0 dBi. It also achieves a consistent half-power beamwidth (HPBW) of 70° ± 5°. The simple structure enhances practicality for base station applications. The design ensures compatibility with both 4G and 5G networks. These attributes make it a strong candidate for next-generation base stations.

Huang et al. (Contribution 4) analyze the limitations of traditional array antenna reliability evaluation methods based on the n/k system. Existing approaches overestimate reliability by ignoring the impact of failed T/R modules on performance. To enhance accuracy, a new evaluation method accounts for performance variations caused by T/R failures in different locations. The method calculates reliability by analyzing all available states of the antenna system. For large-scale array antennas, the system is divided into subarrays, each evaluated separately. The minimum failure threshold for each subarray is determined to refine the overall reliability assessment. The proposed model incorporates fault position and quantity, reducing overestimation errors. Simulation results validate improved accuracy over conventional methods. The approach ensures a more precise reliability assessment for array antennas. This methodology is beneficial for large-scale antenna systems requiring high reliability.

Mohan et al. (Contribution 5) design a zero-order resonant antenna using the composite right-left-handed (CRLH) principle at 30 GHz. The antenna is fabricated without metallic vias, utilizing mirror-image CRLH structures for patch-like radiation. Its characteristics are analyzed through equivalent circuit modeling, parameter extraction, and dispersion diagrams. The measured realized gain is 5.35 dBi, with a radiation efficiency of 87%. The antenna operates without spurious resonance over a 10 GHz bandwidth. A passive beamforming array is implemented using a Butler matrix and CRLH antennas. The CRLH antennas are connected to the 4 × 4 Butler matrix output ports. Beam-scanning angles of 12°, −68°, 64°, and −11° are achieved. Experimental results confirm the antenna’s effectiveness for 5G, wireless power transfer, and IoT sensing. The design offers a compact, high-efficiency solution for next-generation wireless applications.

Dmitriev et al. (Contribution 6) propose a novel graphene antenna with a dipole and four graphene sheet reflectors. The antenna achieves dynamic beam control through electric field-tuning of the chemical potentials of its graphene elements. It supports quasi-omnidirectional, one- or two-directional beam configurations and 360° azimuthal beam steering. An additional graphene layer allows control of the radiation pattern in the elevation plane. The operating frequency range is 1.75–2.03 THz, with a gain varying between 0.86 and 1.63, depending on the active regime. Group-theoretical analysis predicts radiation properties, reducing computational complexity. The proposed antenna offers seamless and continuous beam steering, enhancing adaptability for THz communication. Applications include tracking moving transmitters, medical imaging, and security screening. Numerical simulations validate the theoretical model and performance. Future work will focus on optimizing radiation efficiency and expanding its potential applications.

Farasat et al. (Contribution 7) address pattern distortion in multiband base-station antennas caused by common-mode (CM) currents. The study focuses on mitigating CM interference between low-band (690–960 MHz) and high-band (1810–2690 MHz) antenna elements. A novel common-mode suppression circuit is integrated into the high-band impedance matching network. This circuit shifts the CM resonance frequency outside the low-band range, reducing unwanted distortions. Experimental results confirm significant improvement in low-band radiation patterns. The suppression technique minimizes inter-cell interference and improves network performance. A quarter-wavelength short line with a capacitor effectively suppresses CM currents. High-band performance remains unaffected, maintaining proper impedance matching. The proposed approach ensures stable low-band beamwidth variation within 65° ± 5°. This solution enhances multiband antenna efficiency for 5G base stations.

Ali et al. (Contribution 8) presents a compact ultra-wideband (UWB) antenna with simple geometry. The antenna is designed using the flexible ROGERS 5880 substrate, which has a low dielectric loss and a thickness of 0.254 mm. By modifying a conventional rectangular monopole with triangular slots and a semi-circular stub, the impedance bandwidth is significantly extended. The optimized design covers a wide frequency range of 2.73–9.68 GHz. It features a compact 15 × 20 mm² size, making it ideal for integration into flexible electronics. The antenna maintains stable performance when bent along the x and y axes. Simulated and measured results confirm its robustness in both rigid and flexible conditions. It exhibits an omnidirectional radiation pattern with a minimum gain of >2.5 dBi. The CPW feeding technique ensures easy integration with electronic circuits. The proposed UWB antenna is a strong candidate for flexible and compact wireless applications.

Prado (Contribution 9) presents two near-field models for analyzing spatially fed planar array antennas. The first model is based on radiation equations derived from the A and F vector potentials, while the second model employs the superposition of far-field contributions from individual array elements. Despite different assumptions, both models show a high degree of agreement, with a relative error below 3.2% at 13 λ. The faster model is then used for near-field beam-shaping optimization in a 5G mmWave indoor network. A phase-only synthesis (POS) is applied first to optimize reflectarray layout at 28 GHz. Multi-frequency optimization follows using a method of moments based on local periodicity (MoM-LP). The optimization approach ensures compliance across the 5G NR n257 band. The final optimized antenna achieves a magnitude ripple lower than 1.5 dB. This demonstrates the effectiveness of the proposed method for wideband near-field beam shaping in spatially fed arrays.

Im et al. (Contribution 10) propose a 5G mmWave glass antenna that can be printed on thick vehicle windows. The proposed design features a coplanar waveguide (CPW), a printed monopole, parasitic elements, a linearly arrayed patch director, and a grid-slotted patch reflector. The director and reflector improve bore-sight gain, enhancing the antenna’s high-gain performance. Fabrication and measurements validate broadband operation from 24.1 GHz to 31.0 GHz, with a measured reflection coefficient of −33.1 dB and a peak gain of 6.2 dBi at 28 GHz. Simulation and experimental results exhibit strong correlation. The antenna maintains a 4.5 dBi bore-sight gain even with increasing glass window size. It does not require structural remodeling of the vehicle. The results confirm its suitability for 5G wireless communication.

Odiamenhi et al. (Contribution 11) explore the role of RF energy harvesting in powering IoT and 5G systems for Industry 4.0, highlighting the need for energy-efficient, maintenance-free technologies. The review focuses on challenges in miniaturization, circular polarization, and efficiency, with an emphasis on improving rectifier nonlinearity. It evaluates components like rectifiers, impedance-matching networks, and antennas, particularly in biomedical and IoT applications. Key challenges include rectifier performance, miniaturization trade-offs, and impedance matching. Recommendations include developing versatile rectifiers, advancing miniaturization techniques, optimizing impedance matching, and integrating machine learning for real-time design adjustments. The study concludes that addressing these challenges will enhance the sustainability and efficiency of future IoT and 5G systems.

Collectively, these contributions offer a comprehensive perspective on the latest advancements in antenna design and optimization, specifically for 5G, 6G, and IoT applications. They delve into the multifaceted challenges and opportunities in developing antennas that cater to the ever-expanding demands of modern wireless communication. From the innovative use of graphene in dynamic beam-steering antennas to the integration of RF energy harvesting technologies for sustainable IoT networks, the research highlights both novel solutions and critical considerations in antenna performance, efficiency, and miniaturization. These findings not only push the boundaries of current antenna capabilities but also pave the way for next-generation systems that will support increasingly complex communication networks. The methodologies presented play a pivotal role in shaping the future of wireless communication and sensing, contributing significantly to the evolution of smarter, more efficient, and versatile antenna systems for 5G, 6G, and beyond.

## 3. Conclusions

The journey through the diverse and impactful contributions in this collection has highlighted the breadth and depth of contemporary research in antenna design and optimization for 5G, 6G, and IoT applications. Each contribution, whether focused on novel antenna architectures, innovative optimization methods, or materials advancements, underscores the remarkable progress made in wireless communication and sensing systems. The research presented here not only addresses existing challenges but also pushes the boundaries of what is possible, laying the groundwork for future developments in the field. Collectively, the contributions presented in this issue serve as a powerful testament to the ingenuity and dedication of researchers, each striving to advance antenna technologies in novel directions. From flexible and compact antennas for mobile applications to energy-efficient RF harvesting solutions for IoT devices, the solutions proposed here are shaping the trajectory of future wireless networks and communications. The fusion of advanced materials, miniaturization techniques, and cutting-edge computational methods signifies the vibrant and dynamic nature of antenna research, which will undoubtedly play an even more pivotal role as we move towards an interconnected world driven by 5G and beyond. As we look to the future, new opportunities emerge in enhancing antenna adaptability, improving energy efficiency, and expanding operational bandwidth to meet the demands of emerging technologies. Advancements in machine learning and AI for antenna optimization, alongside the development of sustainable materials, will continue to be at the forefront of this rapidly evolving field. The integration of these technologies will likely revolutionize how antennas function, both in terms of performance and application scope. The future promises exciting challenges and opportunities to further enhance the capabilities of antennas to meet the growing demands of global connectivity, smart cities, and autonomous systems.

A sincere acknowledgment goes to the dedicated editorial board and the supportive editorial office of MDPI’s *Sensors* journal. Their consistent guidance, expertise, and tireless assistance throughout the entire publication process have been indispensable in bringing this collection to fruition. Their unwavering support has significantly contributed to the quality and success of this Special Issue, ensuring that it meets the highest standards of academic excellence. We deeply appreciate their dedication to facilitating the progress and dissemination of cutting-edge research in the field of antenna design and optimization. We are confident that readers will find these contributions both informative and thought-provoking, offering valuable insights into the rapidly advancing world of antenna technologies. As the field continues to evolve, we look forward to further collaborations and future contributions from researchers working to push the boundaries of antenna science. Together, we will continue to advance this field, shaping the future of wireless communications.

## References

[1] Kulkarni P. (2023). Antennas for IoT.

[2] Ojaroudi Parchin N. (2022). Antenna Design for 5G and Beyond.

[3] Ziolkowski R.W. (2022). Electrically Small Antenna Advances for Current 5G and Evolving 6G and Beyond Wireless Systems. Antenna and Array Technologies for Future Wireless Ecosystems.

[4] Parchin N.O. (2023). Editorial on “Design, Analysis, and Measurement of Antennas”. Appl. Sci..

[5] Wu Q., Wang H., Hong W. (2023). Machine Learning-Assisted Optimization and Its Application to Antenna and Array Designs. Advances in Electromagnetics Empowered by Artificial Intelligence and Deep Learning.

[6] Hong W., Jiang Z.H., Yu C., Hou D., Wang H., Guo C., Hu Y., Kuai L., Yu Y., Jiang Z. (2021). The Role of Millimeter-Wave Technologies in 5G/6G Wireless Communications. IEEE J. Microw..

[7] Sharawi M.S. (2014). Printed MIMO Antenna Engineering.

[8] Shi D. (2022). An Intelligent Antenna Synthesis Method Based on Machine Learning. IEEE Trans. Antennas Propag..

[9] Zheng T.-X., Wang H.M., Ng D.W.K., Yuan J. (2019). Multi-Antenna Covert Communications in Random Wireless Networks. IEEE Trans. Wirel. Commun..

